# A three-dimensional computed tomography angiography study of the anatomy of the accessory middle colic artery and implications for colorectal cancer surgery

**DOI:** 10.1007/s00276-020-02511-w

**Published:** 2020-06-04

**Authors:** Mitsuhiro Yano, Shinji Okazaki, Ichiro Kawamura, Shunichiro Ito, Shintaro Nozu, Yuya Ashitomi, Takefumi Suzuki, Yukinori Kamio, Osamu Hachiya

**Affiliations:** Department of Gastroenterological, General, Breast and Thyroid Surgery, Faculty of Medicine, Yamagata University, Yamagata, 990-9585 Japan

**Keywords:** Colorectal cancer, Laparoscopic surgery, Middle colic artery, Transverse colon, Inferior mesenteric artery, Splenic flexure

## Abstract

**Purpose:**

In the present study, we focused on the accessory middle colic artery and aimed to increase the safety and curative value of colorectal cancer surgery by investigating the artery course and branching patterns.

**Methods:**

We included 143 cases (mean age, 70.4 ± 11.2 years; 86 males) that had undergone surgery for neoplastic large intestinal lesions at the First Department of Surgery at Yamagata University Hospital between August 2015 and July 2018. We constructed three-dimensional (3D) computed tomography (CT) angiograms and fused them with reconstructions of the large intestines. We investigated the prevalence of the accessory middle colic artery, the variability of its origin, and the prevalence and anatomy of the arteries accompanying the inferior mesenteric vein at the same level as the origin of the inferior mesenteric artery.

**Results:**

Accessory middle colic artery was observed in 48.9% (70/143) cases. This arose from the superior mesenteric artery in 47, from the inferior mesenteric artery in 21, and from the celiac artery in two cases. In 78.2% (112/143) cases, an artery accompanying the inferior mesenteric vein was present at the same level as the origin of the inferior mesenteric artery; this artery was the left colic artery in 92, the accessory middle colic artery in 11, and it divided and became the left colic artery and the accessory middle colic artery in 10 cases.

**Conclusion:**

3D CT angiograms are useful for preoperative evaluation. Accessory middle colic arteries exist and were observed in 14.9% of cases.

## Introduction

In colorectal cancer surgery, it is essential to dissect the regional lymph nodes to an extent proportional to the stage of the tumor being resected [29]. It is exceedingly important to identify the feeding vessels of the tumor so that the region to be dissected can be determined. The use of laparoscopic navigation in colorectal cancer has been increasing in recent years with the popularization of laparoscopic surgery. Several studies report the advantages of laparoscopic surgery, such as reduced pain because of a smaller incision, earlier recovery of peristalsis, and shorter hospital stays [11, 27]. Laparoscopic surgery also enables more precise manipulation owing to magnification and closer proximity. Still, the narrow field of view and lack of tactile feedback remain problems to be solved. We believe that in order to perform colorectal cancer surgery safely and effectively with curative intent, it is extremely important to determine the arterial branching pattern before commencing surgery. With the rapid progress in high-speed, high-resolution computed tomography (CT) technology and image reconstruction software, it is now possible to quickly see the branching patterns of arteries while keeping invasiveness to a minimum. The superior mesenteric artery, the inferior mesenteric artery, and the vessels supplying the colon and rectum have various anatomical variants, and many studies have reported on this subject [2]. In the present study, we focused on the accessory middle colic artery, which has gained attention in recent years. It has been suggested that investigating the course and branching patterns of this artery, which supplies the left transverse colon, may enable lymph node dissection for cancer of the left transverse colon to be performed more safely and effectively.

### Subjects and methods

A total of 211 cases underwent surgery for neoplastic lesions of the large intestine at the First Department of Surgery of Yamagata University Hospital during the 3-year period between August 2015 and July 2018. Although the hospital, as a rule, performs routine contrast-enhanced CT scans for neoplastic lesions of the large intestine, we excluded cases in which radiographic contrast could not be used due to impaired renal function, or which were difficult to evaluate. We ultimately chose 143 cases for a retrospective investigation. Table 1 shows the patient backgrounds of the 143 subjects. The mean age was 70.4 ± 11.2 years, and there were 86 males and 57 females. Of the 143 cases, 138 were of colorectal cancer, and five were of benign tumor of the large intestine. Laparoscopic surgery had been conducted in 97 of the cases (67.8%).

**Table 1:** Patient background (*n* = 143)

Parameter	*n*
Age^a^ (years)	70.4 ± 11.2
Sex	
Male/female	86/57
Tumor localization	
Vermiform appendix/cecum/ascending colon/transverse colon/descending colon	1/16/29/17/4
Sigmoid colon/rectosigmoid/upper rectum/lower rectum	27/16/19/14
Surgical procedure	
Ileocecal resection/right hemicolectomy/left hemicolectomy	32/26/2
Partial colectomy/sigmoid colectomy/high anterior resection	6/20/13
Low anterior resection/proctectomy/Hartmann’s procedure	20/09/2012
Radical large bowel resection/total pelvic exteneration	01/2
Method of attainment	
Laparotomy/laparoscope	46/97
Level of lymph node dissection	
D0^b^/D1^c^/D2^d^/D3^e^	1/10/56/76
Pathological diagnosis	
Malignant/benign	138/5
pStage	
Benign or 0/I/II/IIIa/IIIb/IV	11/41/42/29/9/11

^a^Mean value ± standard deviation

^b^D0: none

^c^D1: dissection of the peri-intestinal lymph nodes

^d^D2: dissection of the peri-intestinal lymph nodes and intermediate lymph nodes

^e^D3: dissection of the regional lymph nodes

The CT scanner used was an Aquilion ONE (320 slices; Canon Medical Systems). The scan protocol was 120 kVp tube voltage, tube current set to “CT Auto Exposure Control”, 80 slices, 0.5 mm slice thickness, and a helical pitch of 65. One hundred milliliters of a high-density contrast agent was administered intravenously at a rate of 3–4 ml/s. Bolus tracking (Canon Real Prep™) was used to determine the timing for the arterial phase scan. The region of interest was the abdominal aorta at the level of bifurcation of the celiac artery, and imaging was initiated 10 s after a threshold value of 80 HU above the CT value prior to the introduction of contrast was reached.

**Fig. 1:**
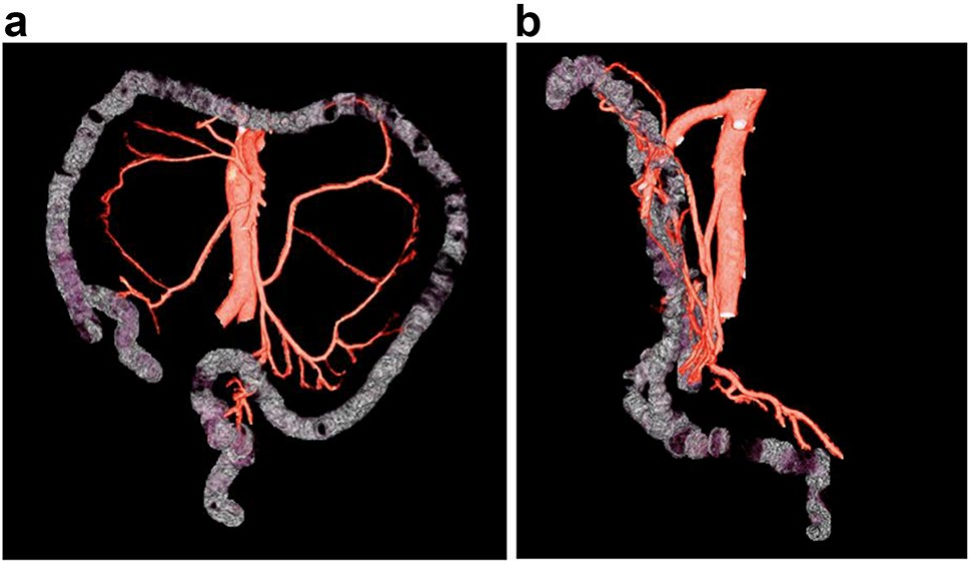
Fusion images of the large intestine and blood vessels. **a** Anterior view. **b** Lateral view

The authors personally processed the image data and traced the blood vessels and the course of the large intestine using the SYNAPSE VINCENT 3D image analysis system (Fujifilm). The large intestine was traced using free hand tracing, and only its course was traced. The blood vessels were traced to the marginal artery as much as possible. Fusion images (Fig. 1) were then made and studied with respect to the items below. The branching patterns and course of the arteries were assessed by three intestinal surgeons, each having over 10 years’ experience, and as part of the assessments, the images were verified in two dimensions in the horizontal and frontal planes.

1. We defined an accessory middle colic artery as “an artery that passes caudally to the lower margin of the pancreas to the distal transverse colon (splenic flexure)”, and sought to find the frequency of its occurrence. We also investigated the variability of its origin.
2. We reconstructed the main trunk of the inferior mesenteric veins and fused them with the artery images; we then sought to find the frequency with which an artery accompanies the inferior mesenteric vein at the same level as the origin of the inferior mesenteric artery. We also investigated which artery accompanied it.

### Ethical considerations

The present study was approved by the Ethics Committee of Yamagata University School of Medicine (FY2018 #287).

## Results

### The accessory middle colic artery

**Fig. 2:**
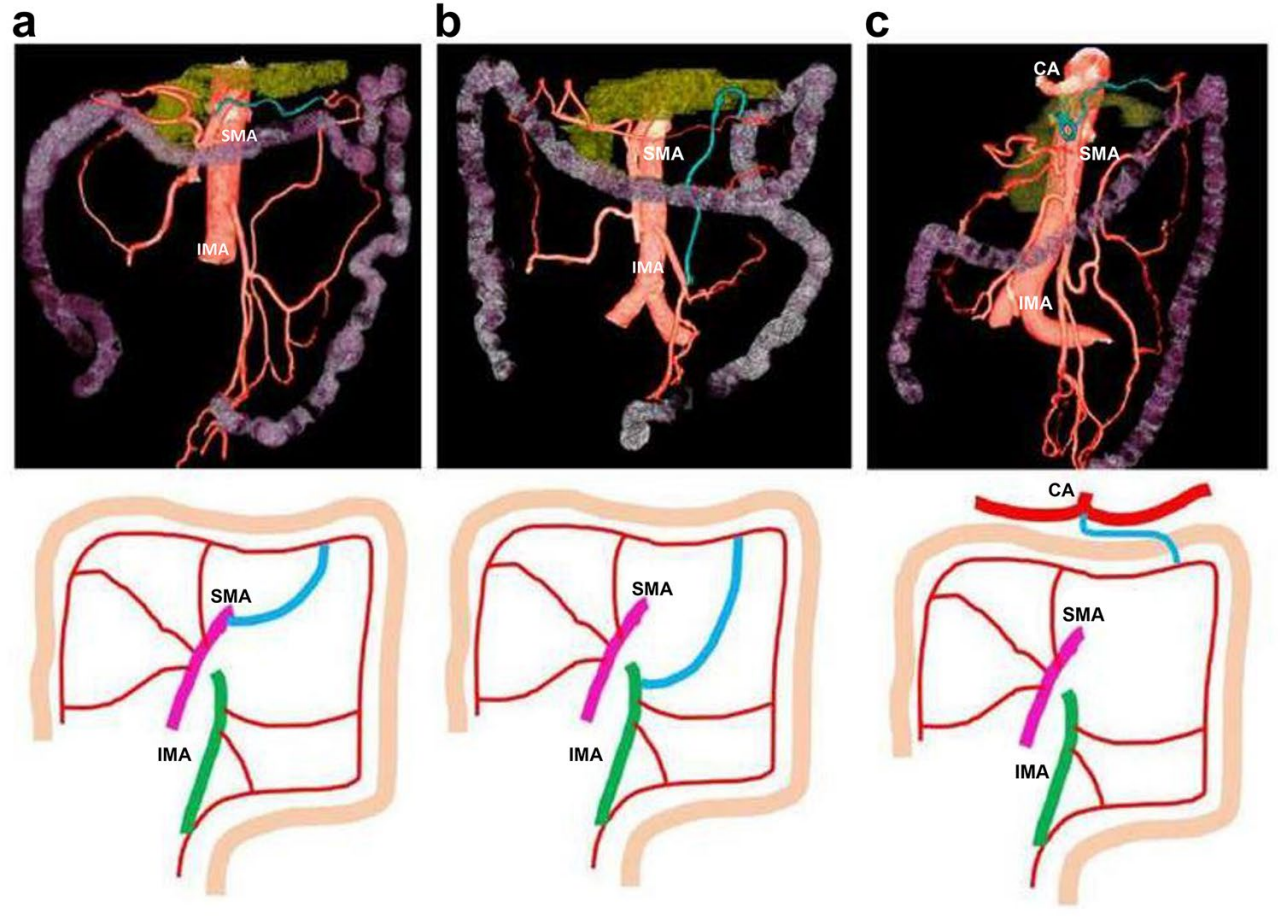
Accessory middle colic artery (blue line), present in 48.9% of cases (70/143). **a** Arising from the superior mesenteric artery: 47 cases (67.1%; 32.9% of total). **b** Arising from the inferior mesenteric artery: 21 cases (30%; 14.9% of total). **c** Arising from the celiac artery: two cases (2.9%; 1.4% of total). *SMA* superior mesenteric artery, *IMA* inferior mesenteric artery, *CA* celiac artery

This was defined as an artery that passed caudally to the lower margin of the pancreas to the distal transverse colon (splenic flexure), and its presence was observed in 48.9% of cases (70/143). It arose from the superior mesenteric artery in 47 cases (67.1%; 32.9% of the total) (Fig. 2a), from the inferior mesenteric artery in 21 cases (30%; 14.9% of the total) (Fig. 2b), and from the celiac artery in two cases (2.9%; 1.4% of the total) (Fig. 2c). Typical examples and the prevalence of each are shown together with diagrams in Fig. 2.

### The inferior mesenteric vein and accompanying artery

**Fig. 3:**
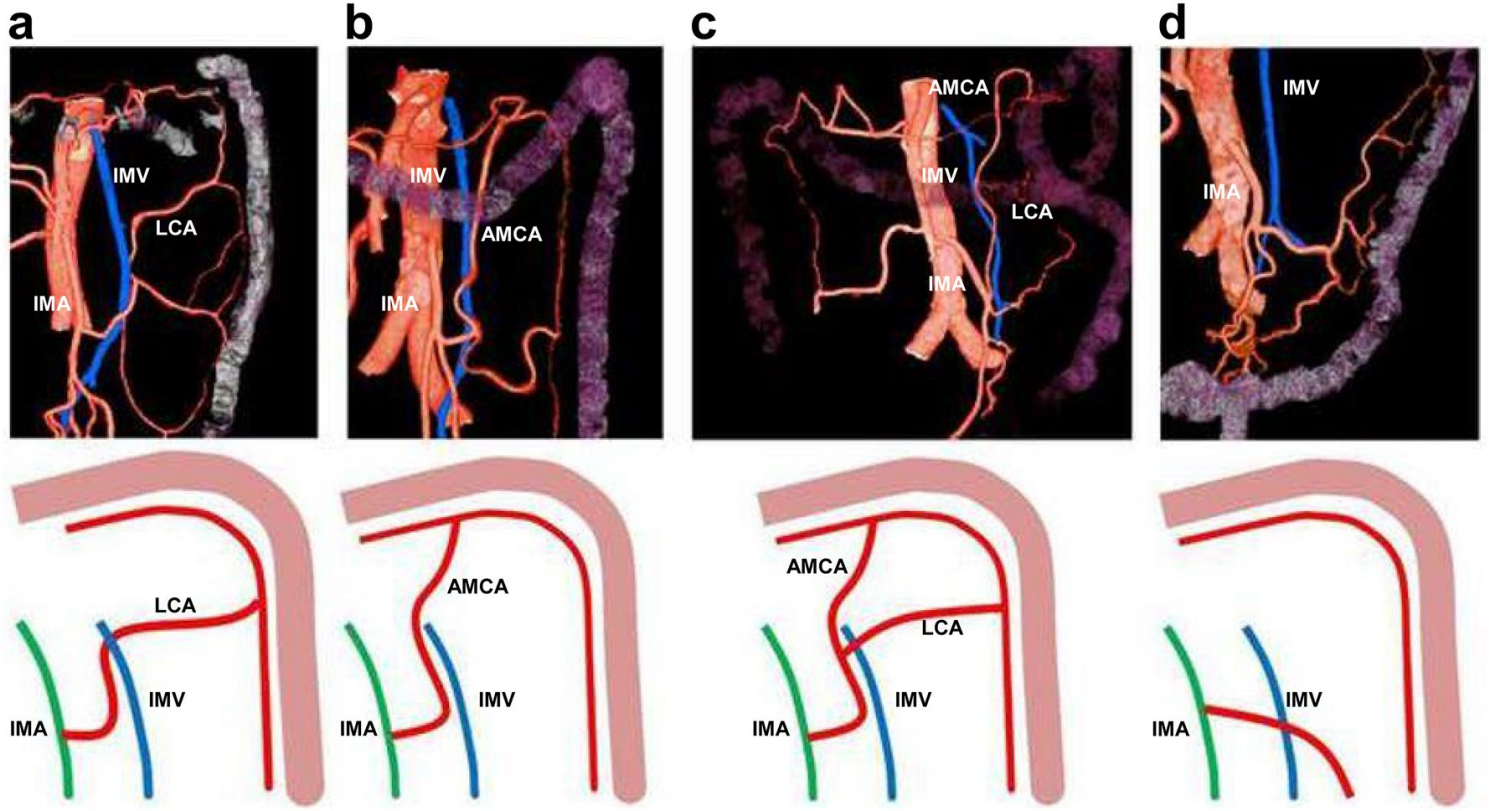
The inferior mesenteric vein and accompanying artery. **a** Type A-1 (accompanied by the left colic artery): 92 cases (81.4%; 64.3% of total). **b** Type A-2 (accompanied by the accessory middle colic artery): 11 cases (9.8%; 7.7% of total). **c** Type A-3 (accompanied by an artery which divides and becomes the left colic artery and accessory middle colic artery): 10 cases (8.8%; 7% of total). **d** Type B (no accompanying artery): 21.8% (31/143). *IMA* inferior mesenteric artery, *IMV* inferior mesenteric artery, *LCA* left colic artery, *AMCA* accessory middle colic artery

We designated cases as Type A when an artery was present that accompanied the inferior mesenteric vein at the same level as the origin of the inferior mesenteric artery, and as Type B when it was not. Type A constituted 78.2% of cases (112/143). The artery was the left colic artery (a blood vessel passing to the proximal descending colon) in 92 cases (Type A-1, 81.4%; 64.3% of the total) (Fig. 3a), the accessory middle colic artery (a blood vessel passing to the splenic flexure of the distal transverse colon) in 11 cases (Type A-2, 9.8%; 7.7% of the total) (Fig. 3b) and in 10 cases (Type A-3, 8.8%; 7% of the total) (Fig. 3c), it later divided and became the left colic artery and the accessory middle colic artery. Type B (no accompanying blood vessel) cases constituted 21.8% (31/143) of the total (Fig. 3d).

## Discussion

The basic idea in surgical oncology is that in cancer surgery, especially lymph node dissection, an artery is ligated and resected at its origin, and all the associated organs, adipose tissue, and connective tissue, are removed en bloc, together with the related lymph nodes. Therefore, it is extremely important to investigate the course of arteries before commencing surgery. In colorectal cancer surgery, a feeding vessel, defined by the Japanese classification system as an artery that exists within 10 cm of a tumor, is to be resected at its origin to remove the regional lymph nodes [10]. In colorectal surgery, the variation in the branching patterns of the arteries of the large intestine must be considered when dissecting the lymph nodes at the origin of the artery, and it is important to verify the course of each artery by mapping the blood vessels before surgery.

There are many reports of studies of the superior mesenteric artery territory alone, or the inferior mesenteric artery territory alone. Some reports detail studies of cadaver dissections and some of angiography [5, 17, 30]. Recently, with the invention of imaging devices, the number of reports in which studies were conducted with images of blood vessels constructed from CT scans taken before surgery, is increasing [7, 13, 16]. There are also various reports about the agreement between preoperative CT vascular reconstructions and surgical findings of resected specimens [8, 9, 19, 23]; Hoshino et al. reported that morphological characteristics observed in preoperative CT scans were consistent with those observed in the actual resected specimens in each of the seven cases that they studied [8], and Nesgaard et al. reported that diagnostic accuracy was 97.1%, sensitivity was 85.7%, and specificity was 95.2% [19]. However, most of the reports until now have only categorized the patterns by which vessels branch off the trunks of the superior and inferior mesenteric arteries, and there are few studies in which the vessels are traced out to their periphery. In the present study, we mapped the course of the entire large intestine and its related blood vessels as far as possible out to their periphery, and conducted investigations.

In previous reports, the accessory middle colic artery has been defined as “an artery that arises from the proximal part of the superior mesenteric artery and passes to the splenic flexure of the transverse colon after passing to the lower margin of the pancreas”. Considering the region that it supplies, we believe that it would be more understandable if it were called the “transverse colon splenic flexure artery”.

In the present study, we traced the arteries up to the marginal artery while considering the course of the colon and defined the accessory middle colic artery as “an artery that passes caudally across the lower margin of the pancreas to the distal transverse colon (splenic flexure)”. Further, we distinguished it from the left colic artery, which was defined as “a vessel arising from the inferior mesenteric artery and passing to the descending colon”. This enabled identification of an accessory middle colic artery in 48.9% (70/143) of cases.

The origin of the artery was in the superior mesenteric artery in 47 cases (67.1%; 32.9% of the total), in the inferior mesenteric artery in 21 cases (30%; 14.9% of the total), and in the celiac artery in two cases (2.9%; 1.4% of the total). As previous studies report that it exists in 4–8% of cases in the USA and Europe, and in 33–49% of cases in Japan [1, 4, 7, 15, 20, 21, 28, 30], its frequency of occurrence in the present study (48.9%) is high. Moreover, although there are reports of an accessory middle colic artery that arises in the inferior mesenteric artery, it occurs infrequently, appearing in 5–9% of cases (30% in the present study) [20, 30]. We believe that the results of the present study indicate that part of the ascending vessel arising from the inferior mesenteric artery that was previously thought to be the left colic artery that supplied the descending colon is a vessel that passes the splenic flexure of the transverse colon.

Although cancer occurs rarely in the splenic flexure of the transverse colon, it frequently leads to blockages and/or distant metastases, and prognosis is generally considered to be poor because surgery is difficult [18, 24]. It is widely held that the left branch of the middle colic artery is the blood vessel that supplies the left side of the transverse colon. However, we believe that it is important to be aware of the accessory middle colic artery (and especially of the vessels that arise in the inferior mesenteric artery), which is not uncommon, and to assess, before beginning surgery, which territories should be resected. However, the situation is not completely clear with regard to lymph flow at the left side of the transverse colon, including in cases in which an accessory middle colic artery arises in the inferior mesenteric artery, and further study will be required in the future.

When a D3 dissection (i.e., dissection of regional lymph nodes) is performed during surgery for cancers of the distal sigmoid colon and rectum, the inferior mesenteric artery is resected at its origin, together with the left colic artery, which ascends beside it at the same level. Consequently, it is important to know whether another artery accompanies the inferior mesenteric vein at the same level as the origin of the inferior mesenteric artery, and if does is, whether the accompanying artery is truly the left colic artery. In the present study, an accompanying artery was present in 78.2% (112/143) of cases. This result resembles one in a study by Ke et al., who reported that the left colic artery was close to the inferior mesenteric vein in 71% of cases [14]. However, in their report, any ascending artery was counted as the left colic artery. In the present study, that artery was the left colic artery (a blood vessel passing to the proximal descending colon) in 92 cases (81.4% of the instances in which an artery was present; 64.3% of the total), the accessory middle colic artery (a blood vessel passing to the splenic flexure of the distal transverse colon) in 11 cases (9.8% of instances; 7.7% of the total) and in 10 cases (8.8% of instances; 7% of the total), it later divided and became the left colic artery and the accessory middle colic artery. This is the first study that investigates areas supplied by arteries that are resected when performing D3 dissections. There is an artery called the arc of Riolan, named after the French anatomist Jean Riolan, which runs between the left and middle colic arteries. This artery is reported to exist in 3.2–8.9% of cases [12]. The 7% of cases in the present study in which the ascending artery divided and became the left colic artery and accessory middle colic artery would be included among such cases. In a study of 191 cases in which the blood supply to the splenic flexure was evaluated, there was only one case in which the arc of Riolan connected these two marginal arteries [4]. We believe it is possible that part of the arc of Riolan mentioned in that study was the same as the vessel described in the present study that connects the left colic artery and accessory middle colic artery.

Suture failure is the biggest complication of surgery for colorectal cancer in the distal side of the sigmoid colon. It has been reported that anastomotic bleeding is decreased if the inferior mesenteric artery is resected at its origin [3, 22]. It is not yet clear whether procedures that preserve the left colic artery contribute to a reduction in suture failure rates [6, 25, 26]. We believe, however, that when lymph node dissection must be performed in a way that prioritizes the preservation of blood flow to the anastomosis, it is important to understand not only the branching patterns of the inferior mesenteric artery but also which areas are supplied by the arteries being preserved. It is surgically difficult to perform a D3 dissection that preserves the left colic artery when there is no ascending left colic artery. Furthermore, we believe that when the ascending artery is the accessory middle colic artery, as it runs for a long distance before joining with the marginal artery, there is the possibility that an increase in blood flow at the oral margin (the descending colon or the sigmoid colon) caused by preserving the artery, will be localized.

## Conclusion

Studying blood vessels before surgery using 3D CT angiograms is minimally invasive and can be performed at most hospitals. An accessory middle colic artery branching off the inferior mesenteric artery was present in 14.9% of cases. We believe that it is important to conduct preoperative assessments that take into consideration the existence of this blood vessel before beginning surgery for colorectal cancer, particularly in cases of left-sided colorectal cancer.
